# Activity Tracking Devices in Group Prenatal Care: A Feasibility Study

**DOI:** 10.1089/biores.2018.0021

**Published:** 2018-11-28

**Authors:** Michelle A. Kominiarek, Heidi Vyhmeister, Lauren C. Balmert, Paige Fairchild, Hallie Tolo, William Grobman, Melissa Simon

**Affiliations:** ^1^ Division of Maternal-Fetal Medicine, Department of Obstetrics and Gynecology, Northwestern University, Chicago, Illinois.; ^2^ Department of Women's Health, Erie Family Health Center, Chicago, Illinois.; ^3^ Division of Biostatistics, Department of Preventive Medicine, Northwestern University, Chicago, Illinois.; ^4^ Department of Obstetrics and Gynecology, Northwestern University, Chicago, Illinois.

**Keywords:** activity tracking devices, pregnancy, physical activity, group prenatal care, feasibility study

## Abstract

To evaluate the feasibility (adherence to the study protocol and satisfaction) of using an activity tracking device (ATD) in group prenatal care. Women participated if they (1) were in group prenatal care, (2) owned a smartphone, and (3) had no activity restrictions. Women were instructed to wear and sync the ATD daily. Protocol adherence and satisfaction were assessed via surveys. Mixed models assessed the relationship between gestational age and ATD data. Self-reported energy expenditure from the Pregnancy Physical Activity Questionnaire (PPAQ) was compared with ATD-calculated energy expenditure. The baseline characteristics of the 49 women were as follows: 24 years old, prepregnancy body mass index 28, 80% Hispanic, 86% nulliparas, and 21 weeks of gestation. Of the 30 women who completed the follow-up survey, 47% self-reported wearing the ATD daily, 27% reported a lost or broken ATD, and 22% reported technical problems; however, 97% enjoyed wearing it, 100% would recommend it to a pregnant friend, and 77% thought it helped them reach activity goals. According to ATD data, the median active days were 47 (interquartile range [IQR] 21–79) and the median proportion of active days of potential days was 43.7% (IQR 15.4–77.1). For women who wore the ATD for the first 7 days, mean steps/day were 7574 (range 3076–15,828), active minutes/day were 277 (range 145–475), and sedentary hours/day were 12 (range 7.8–16.2). As gestational age increased, mean log steps decreased, mean active minutes decreased, and mean sedentary hours increased in unadjusted and adjusted models (*p* < 0.001 all comparisons). There were no differences in mean energy expenditure (MET-h/week) by PPAQ or ATD data at 28 weeks of gestation [231 (62–927 range) vs. 238 (212–290 range), *p* = 0.74] and at 36 weeks of gestation [145 (35–581 range) vs. 222 (196–272 range), *p* = 0.27]. Most women reported high satisfaction with an ATD in group prenatal care, yet adherence to the study protocol was low and ATD technical problems were common. As gestational age increased, activity decreased while sedentary time increased, suggesting that additional research is needed to find ways to engage women in physical activity during pregnancy.

## Introduction

Physical activity has multiple beneficial health effects that include reducing the risk of gestational diabetes, maintaining physical fitness, and enhancing psychological well-being during pregnancy.^1^ Pregnancy is often considered the optimal time to intervene for health behaviors related to eating habits and physical activity so as to prevent excessive gestational weight gain (GWG). In support of health behavior interventions for GWG, a meta-analysis of 49 randomized controlled trials with 11,444 women reported that diet or exercise interventions during pregnancy reduced the frequency of excessive GWG by 20% (RR 0.8, 95% CI 0.73–0.87).^2^

Since it can be difficult to enhance physical activity during pregnancy, such an intervention needs to be dynamic and flexible to account for the barriers that accompany this challenging life stage. The capabilities for self-monitoring are now more contemporary than older versions such as exercise logs, diaries, or pedometers. Using validated questionnaires to measure physical activity is simple and inexpensive, yet these tools have limitations due reliability and construct validity.^3,4^ Furthermore, other studies suggest that there is only fair agreement in self-reported physical activity using questionnaires and objectively measured physical activity (e.g., pedometers, accelerometers) and that self-reported activity is typically higher than what accelerometers record.^5,6^

Text messaging and m-health apps (apps) can facilitate engagement and retention in physical activity interventions. In addition, self-monitoring, an important construct for long-term behavior change is already built into the app technology.^7^ Activity tracking devices (ATDs) are newer ways to objectively assess physical activity by providing data about steps taken, distance traveled, and energy expenditure or calories burned, but have been rarely evaluated in pregnancy.

Compared with traditional prenatal care, group prenatal care models have been designed to improve patient education and include opportunities for social support by bringing patients with similar needs together for health care. Self-monitoring in the form of weight and blood pressure monitoring is a component of many group prenatal care models. Evidence suggests that women in group prenatal care have improved prenatal knowledge, greater pregnancy-related empowerment, feel more ready for labor and delivery, and are more satisfied with their care.^8,9^ Participation in group prenatal care may also improve perinatal outcomes and contributing health behaviors.^10^ However, the evidence for GWG in group prenatal care is mixed with some analyses showing a reduction in the frequency of excessive GWG,^11–13^ and other studies suggesting no association between excessive GWG and the type of prenatal care model.^14–16^

Due to the potential for group prenatal care to have salutary effects on health behaviors, the importance of meeting GWG and physical activity goals for a woman's long-term health, and the lack of evidence for ATD in group prenatal care, the primary objective of this study was to evaluate the feasibility (adherence to study protocol and satisfaction) of using a commercially available ATD in group prenatal care, where the embedded social support could motivate women to meet their pregnancy goals, including physical activity. In addition, we also aimed to evaluate the ATD data over the course of pregnancy and compare self-reported with ATD-measured energy expenditure.

We hypothesized that participants would wear the ATD for more than 80% of the time from enrollment until delivery and that less than 10% of participants would report major issues or technical difficulties with the ATD or cell phone. We also hypothesized that self-reported physical activity would differ from the energy expenditure calculated from the ATD.

## Materials and Methods

A large federally qualified health center in Chicago has offered the CenteringPregnancy model of group prenatal care since 2005 at three separate locations to women at their initial intake or first prenatal care visit. The majority of women in CenteringPregnancy are low-income minority nulliparas with a low-risk pregnancy (e.g., absence of diabetes, hypertension, or multiple gestations) at this site.

For this study, pregnant women who were enrolled in group prenatal care at this site were approached at their first or second CenteringPregnancy session and asked to participate in a study about “activity monitoring devices and pregnancy.” Other inclusion criteria were English or Spanish speaking, ≥18 years old, and personal ownership of a smartphone. Exclusion criteria were restrictions or inability to exercise, defined as at least 30 min of walking per day. Prior or current ownership of an ATD was not an exclusion criterion. The study period extended from May 2016 to May 2017.

After written informed consent was obtained, participants picked a wrist Fitbit Flex™ (i.e., the ATD) in their color preference. In-person instructions were given on how to install the ATD app on their smartphone, charge and sync the ATD, wear the ATD, and interpret the ATD data from the dashboard. Members of the research team registered the participants' ATD online and created user accounts authorizing access to the ATD data for the research personnel. The user accounts were available in both English and Spanish, according to the participants' preferences. Participants in the study also received a 10-min in-person group counseling session in English or Spanish on GWG goals specific to their prepregnancy body mass index (BMI), step count goals, and safe exercises during pregnancy.

The exercise recommendations were adapted from the American Congress of Obstetricians and Gynecologists Committee Opinion on “Exercise in Pregnancy.”^17,18^ In terms of step count goals, participants were advised that 10,000 steps/day was one recommendation, but that this goal may not apply to all pregnant women.^19,20^ They were advised to consider the steps in the 7 days of ATD use as their “baseline” and to gradually increase the number of steps (e.g., 500 steps/day) until they reached their own personal goal or 10,000 steps/day. Steps, active minutes, and sedentary hours were wirelessly transmitted via cellular and Bluetooth technology and plotted on a graph in the ATD app that participants could view on their device or personal dashboard at all times (i.e., participants not blinded to data). Of note, the Fitbit Flex does not have heart rate monitoring. Members of the research team contacted the participant via text, phone calls, or e-mail if more than 72 h lapsed since the ATD was synced. In-person visits with the research team also occurred when these contact methods were not successful. Lost, stolen, or broken ATDs were not replaced. The research team provided technical support for the ATD throughout the pregnancy with text messages, phone calls, e-mails, or in-person troubleshooting sessions.

Participants also were asked to complete the following: (1) a baseline survey about their own demographic characteristics and personal technology use; (2) Pregnancy Physical Activity Questionnaires (PPAQs)^21^ at baseline, 28 weeks of gestation, and 36 weeks of gestation; and (3) surveys at 36 weeks regarding change in health behaviors and satisfaction. The changes in health behaviors were assessed via questions about changes in exercise before pregnancy and 36 weeks (e.g., “Before pregnancy, how much did you exercise” and “Since you became pregnant, how much are you exercising?). Satisfaction was assessed with 15 items about benefits and barriers to ATD use, as adapted from another study of ATD use in women (e.g., “I had difficulties wearing the ATD because…,” “I had the following problems with the ATD website…,” “The benefits of wearing the ATD for me were…”).^22^

The PPAQ contains questions about time spent in the current trimester in 32 activities, including household/caregiving, occupational, sports/exercise, transportation, and inactivity. The self-reported time spent in each activity was multiplied by its intensity to measure average weekly energy expenditure (MET-h/week) attributable to each activity.^23^ These surveys were available either via the REDCap system (Research Electronic Data Capture, an online tool for secure data capture) or in paper format, according to participant preference.^24^ Most importantly, participants were counseled to wear the ATD continuously (all day, all night) during the pregnancy and only remove it when it was at risk for damage (e.g., swimming and bathing) or being charged. It was expected that the ATD would by synced every day and charged every 5 days. All participants were enrolled as members of the Fitbit Community Activity Group that was named “CenteringPregnancy Fitbit Study,” so that each could view anonymously all others' activity progress. The participants kept the ATD at the end of the study, at which time the research account was deactivated.

Participants received standard prenatal and postpartum nutrition and exercise counseling from their providers as part of CenteringPregnancy.^18^ The participants' medical records were reviewed to abstract the total number of prenatal visits and CenteringPregnancy sessions attended. If participants opted not to continue in CenteringPregnancy, but continued to receive traditional prenatal care at the site, they continued in the study and their data were collected. The data from the surveys and electronic medical records were eventually merged with the ATD data, which was collected from Fitabase, the research platform for data management with Fitbit.

Participant dropouts and the reasons for any drop out were recorded and summarized. Any technical problems, including loss or malfunction of ATD, charger, or cell phone, were recorded and summarized. Adherence to the ATD intervention was evaluated by the median and interquartile range (IQR) number of days the ATD was worn from the day of study entry until delivery for each participant, defined by a minimum step count of >1000/day.^25,26^

The proportion of active days out of potential days, the longest number of consecutive days worn, and number of participants wearing the ATD for at least seven consecutive days were reported. ATD data, including mean daily steps, active minutes, and sedentary hours, also were summarized for the first full week of use. For the proportion of active days out of potential days, the analysis was restricted to participants who did not report permanent technical problems (e.g., lost or broken ATD or charger, lost cell phone access, unresolved syncing issues). The responses from the health behavior changes and satisfaction surveys were recorded and summarized.

We evaluated the number of steps, active minutes, and sedentary hours per day over the pregnancy period. The Fitbit technology calculates active minutes using metabolic equivalents (METs) and reports them as “very active minutes,” “fairly active minutes,” and “lightly active minutes.” Fitbit MET data were converted to total MET-h/week and averaged over the second and third trimesters. For purposes of this analysis, we combined the active minutes into one group to account for any activity ≥1.5 METs as sedentary time (e.g., seated activities) is typically defined as an activity with less than 1.5 METs.^27^

First, we graphically assessed trends in ATD data by gestational age. Outcomes were averaged over participants who had recorded ATD data at the corresponding gestational age, and local regression (LOESS) curves, a nonparametric method, were fit over the scatter plots to reveal further trends in the data. We then used mixed models to assess the trajectory of each of the three outcomes over the pregnancy period, similar to Huberty et al.^28^ Models included a fixed effect for gestational week and a random effect for participants to account for correlations of measurements within participants. Adjusted models also were considered with inclusion of baseline covariates, including age, ethnicity, prepregnancy BMI, and education. Higher order terms were considered, model assumptions were assessed, and transformations of outcomes were performed as necessary.

To assess changes over time in the PPAQ, a series of paired *t*-tests or Wilcoxon signed rank tests were performed, as appropriate. Mean MET-h/week for the second (up to 28 weeks of gestation) and third trimesters (up to 36 weeks of gestation) also was calculated from ATD data. Energy expenditures as determined from the PPAQ and ATD were compared via Wilcoxon signed rank tests. We further assessed the equality of variance between the two measures using Pitman's test with Spearman correlations.

Our hypotheses that participants would wear the ATD for more than 80% of the time and that less than 10% would report major issues or technical difficulties with the ATD came from a pilot survey at one of our clinical sites with similar patient demographics. Of the 25 women surveyed, 100% had a cell phone, 88% said they would wear the ATD all the time, and 88% said they would not need any technical support using the app or assistance installing the device on their phone. All hypothesis tests assumed a two-sided type 1 error rate of 0.05 and no adjustments were made for multiple comparisons. Analyses were performed with SAS, version 9.4. Because this was a feasibility study, no power calculation was performed. The study was approved by the Northwestern University and the Erie Family Health Center IRB.

## Results

Seventy-four women from a total of 162 women enrolled in CenteringPregnancy were approached to participate in this study during the 13-month recruitment period; 3 were ineligible due to age <18 years or lack of cell phone. Of those who declined, the most common reasons were lack of time to stay for study enrollment procedures (*n* = 3), current ownership of an ATD (*n* = 2), and “just not interested” (*n* = 15). Although 51 women were enrolled in the study, two did not use the ATD because they decided not to participate after consenting, leaving 49 women with at least 1 day of ATD use. Five women later opted out of the study due to either cell phone problems or personal reasons (e.g., “did not fit my lifestyle”), three women were lost to follow-up, and one woman had a miscarriage at less than 20 weeks.

Table 1 presents the maternal demographics and characteristics of the initial 49 women. The majority of the participants identified as Hispanic, but only one participant preferred communication in Spanish. Eighty-six percent of the women were nulliparas, 69% were either overweight or obese, and more than 90% used the Internet daily and reported a high level of comfort using computers. Only 11% of the women reported that they exercised daily before pregnancy. The mean number of CenteringPregnancy and total prenatal care visits was 5.1 ± 2.9 and 12.5 ± 3.0, respectively, for all 49 participants.

**Table 1.** **Maternal Demographics and Characteristics**

Variable	Response
Age, years (mean ± SD)^a^	24.1 ± 4.2
Race/Ethnicity, *n* (%)^b^	
Asian American	2 (4.4)
Black/African American	6 (13.3)
Hispanic/Latino	36 (80.0)
Other	1 (2.2)
Education, *n* (%)^b^	
Grades 9–11	2 (4.4)
High school graduate/GED	17 (37.8)
Some college/technical school	21 (46.7)
Four-year college degree or more	5 (11.1)
Health insurance, *n* (%)^b^	
Medicaid or Medicare	39 (86.7)
Private insurance	2 (4.4)
Other	4 (8.9)
Employed outside of the home for a salary, *n* (%)^b^	
Yes	25 (55.6)
No	20 (44.4)
Marital status, *n* (%)^b^	
Married	11 (24.4)
Single	11 (24.4)
Living with partner, but not married	23 (51.1)
Nullipara, *n* (%)^a^	42 (85.7)
Gestational age at enrollment, weeks (mean ± SD)^b^	21.3 ± 5.1
Trimester at enrollment, *n* (%)^a^	
First	1 (2.0)
Second	42 (85.7)
Third	6 (12.2)
Prepregnancy BMI (mean ± SD)^a^	28.3 ± 6.7
Prepregnancy BMI, *n* (%)^a^	
Underweight	3 (6.1)
Normal	12 (24.5)
Overweight	17 (34.7)
Obese	17 (34.7)
History of regular cigarette use, *n* (%)^b^	
Yes	5 (11.1)
No	39 (86.7)
Unknown	1 (2.2)
Self-reported daily Internet use, *n* (%)^b^	41 (91.1)
Self-reported “very comfortable” using a computer and/or the Internet, *n* (%)^b^	41 (91.1)
Type of smartphone owned, *n* (%)^b^	
iPhone	31 (68.9)
Droid	14 (31.1)
“Before pregnancy, how much did you exercise?” *n* (%)^b^	
Not at all	8 (17.8)
Occasionally	16 (35.6)
Once a month	3 (6.7)
Once a week	4 (8.9)
More than 1 time a week	9 (20.0)
Every day	5 (11.1)
“Before pregnancy, describe your nutrition,” *n* (%)^b^	
Very poor	4 (8.9)
Poor	5 (11.1)
Average	25 (55.6)
Good	11 (24.4)

^a^ Total analytic cohort, *n* = 49.

^b^ Subset of analytic cohort completing baseline survey, *n* = 45.

BMI, body mass index; GED, general equivalency development.

The responses from the 36-week adherence and satisfaction survey are summarized in Tables 2 and 3. Of the 30 participants who completed the 36-week survey, only 46.7% reported wearing the ATD all the time. Some of the reasons for not wearing the ATD included concerns that it would get lost, stolen, or damaged. In addition, most participants did not use the community forum or message boards for technical support (80%) or to view other participants' data (90%). Overall, participants reported ease of use with the ATD and app, but difficulties syncing the tracker with the app were common. The majority of participants enjoyed wearing the ATD (97%), would recommend it to a pregnant friend (100%), and thought that being in the study helped them eat more healthily (80%) and reach their activity goals (76%).

**Table 2.** **Self-Reported Adherence and Changes in Health Behaviors Based on Surveys at 36 Weeks (*n* = 30)**

Variable	Response at 36 weeks, *n* (%)
Adherence	
“How often are you wearing the ATD?”	
All the time	14 (46.7)
A few hours a day	3 (10.0)
Only when I'm awake	3 (10.0)
A few days a week	8 (26.7)
Other	2 (6.6)
“I have difficulties wearing the ATD because”^a^	
Concern that it would get lost or stolen	2 (6.7)
Concern that it would get damaged if it got wet	4 (13.3)
Broken device	1 (3.3)
Other reasons (e.g., forget to charge or wear, moved residence, personal problems, drained cell phone battery)	10 (33.3)
“I had the following problems with the ATD or app.”^a^	
Internet connection problems	3 (10.0)
Too much work to enter information	2 (6.7)
Did not like the website	1 (3.3)
Did not like wearing Fitbit tracker	1 (3.3)
Difficulty getting Fitbit tracker to sync with website	5 (16.7)
Other technical problems with Fitbit tracker	2 (6.7)
Lost or broken Fitbit tracker or charger	8 (26.7)
“What were the benefits of wearing an ATD for you?”^a^	
I knew the number of steps I took per day	21 (70.0)
I learned how my activity varies each day	14 (46.7)
I improved my health by tracking my activities and goals	7 (23.3)
Health behavior changes	
“How much are you exercising since pregnancy?”	
More often	2 (6.7)
About the same	7 (23.3)
Less often	21 (70.0)
“How has your nutrition changed compared with before pregnancy?”	
Improved	13 (43.3)
Stayed the same	16 (53.3)
Worsened	1 (3.3)
“Physical activity that makes me breathe harder is ok at any time during pregnancy.”	1 (3.3) Strongly agree
	14 (16.7) Agree
	12 (40) Disagree
	3 (10) Strongly disagree

^a^ Categories are not mutually exclusive, so percentages do not sum to 100.

ATD, activity tracking device.

**Table 3.** **Self-Reported Satisfaction Based on Surveys at 36 Weeks (*n* = 30)**

Satisfaction questions, *n* (%)	Strongly agree	Agree	Disagree	Strongly disagree
I found the Fitbit website and dashboard easy to navigate	6 (20.0)	22 (73.3)	1 (3.3)	1 (3.3)
I found the smartphone Fitbit app easy to use	16 (53.3)	14 (46.7)	0	0
I enjoyed wearing the Fitbit	9 (30.0)	20 (66.7)	1 (3.3)	0
I would recommend the Fitbit to a pregnant friend	18 (60.0)	12 (40.0)	0	0
Being in this study helped me eat healthier	3 (10.0)	21 (70.0)	6 (20.0)	0
Being in this study helped me reach my activity goals	5 (16.7)	18 (60.0)	7 (23.3)	0
Being in this study helped me reach my weight gain goals	4 (13.3)	13 (43.3)	13 (43.3)	0
I am satisfied with my weight gain this pregnancy	7 (23.3)	15 (50.0)	8 (26.7)	0

Based on pairwise comparisons of PPAQ self-reported total activity during the three time periods, there were no significant differences between any two time points (*p*-value = 0.14 for comparison of baseline vs. 28 weeks, *p*-value = 0.28 for comparison of baseline vs. 36 weeks, *p*-value = 0.79 for comparison of 28 vs. 36 weeks), even though 70% of participants responded that they were exercising less often compared with before pregnancy at the 36-week adherence and satisfaction survey.

**FIG. 1.**
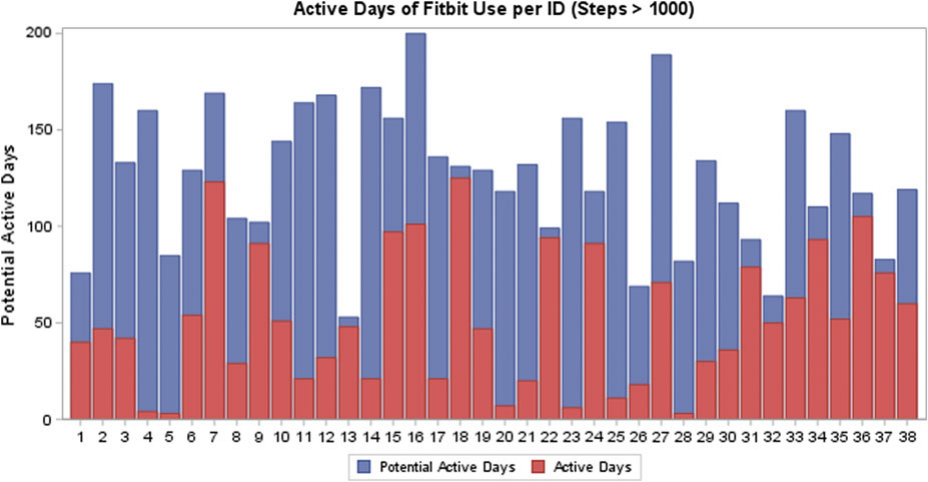
Active days of ATD use, defined as at least 1000 steps/day (red bars), as a proportion of total potential active days (blue bars) from date of enrollment to date of delivery for 38 participants who did not report permanent ATD or cell phone problems (e.g., lost or broken ATD or charger, loss of cell phone access). ATD, activity tracking device.

**FIG. 2.**
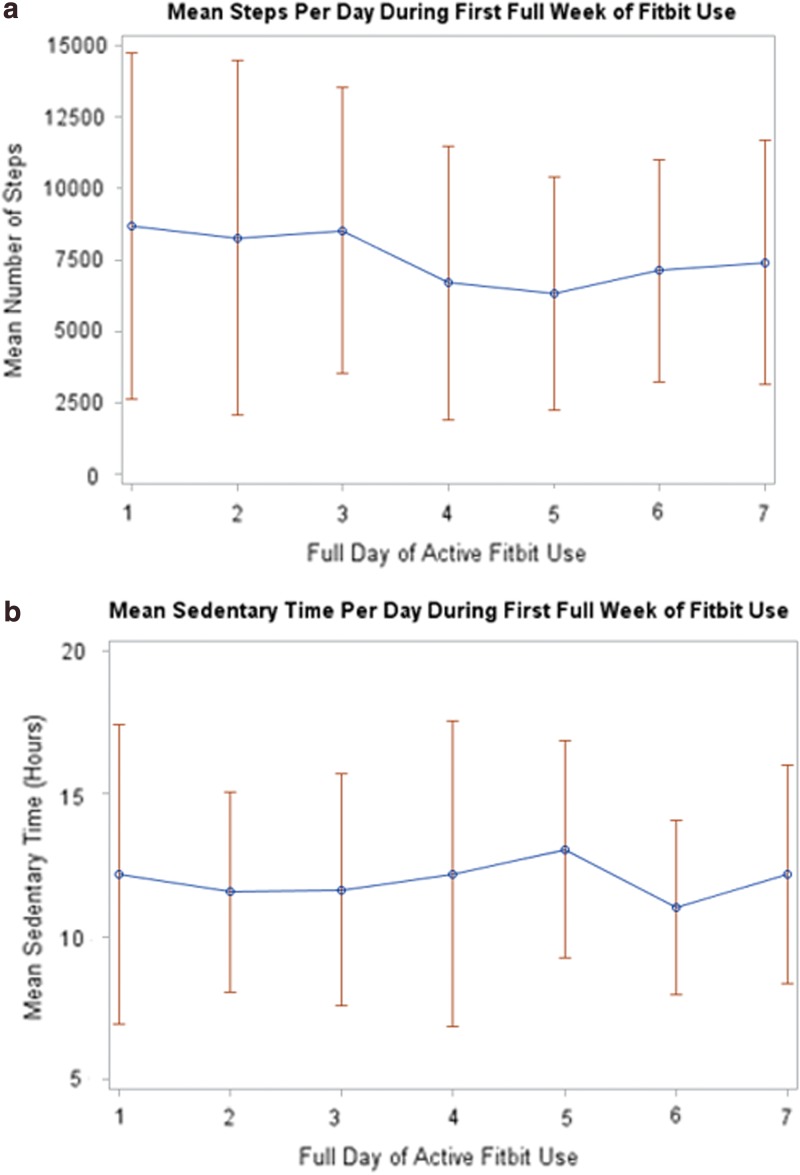
Mean (blue dots) and range (red bars) of **(a)** steps and **(b)** sedentary hours for 25 participants who wore the ATD consecutively for the first 7 days.

According to the ATD data, the median number of active days was 47.5 (IQR 21.0–79.0) and the median proportion of active days was 43.7% (IQR 15.4–77.1) (Fig. 1) for 38 participants who did not lose the ATD or charger and/or encounter unresolved syncing or cell phone difficulties. The median number of days women had the potential to wear the ATD was 130 (IQR 102–156). The longest period of time that a participant wore the ATD was 94 days and 75% of participants wore the ATD for at least seven consecutive days. Of the 25 participants who wore the ATD consecutively for the first full 7 days of enrollment, the mean steps per day during the first week were 7574 (range 3076–15,828), active minutes per day were 277 (range 145–475), and sedentary hours per day were 12 (range 7.8–16.2) (Fig. 2). Twenty percent had <5000 mean steps, 48% had 5000–7499 mean steps, and 32% had ≥7500 mean steps/day.

**FIG. 3.**
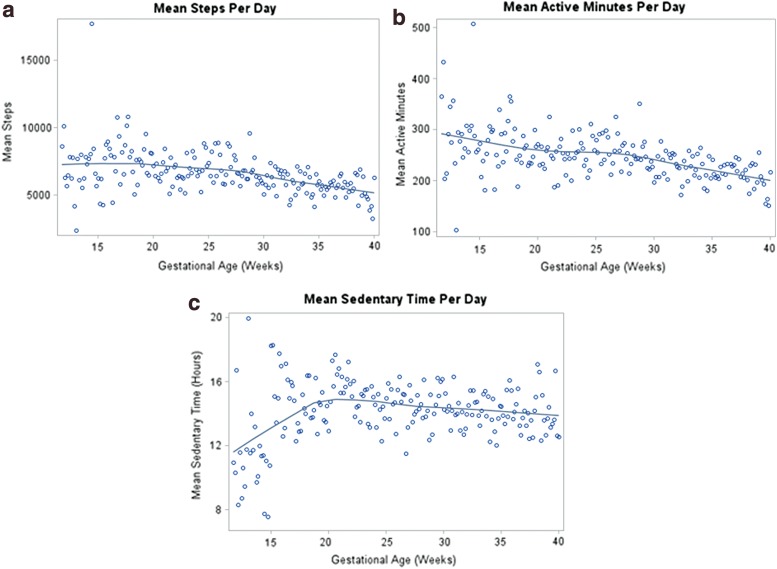
Mean **(a)** steps, **(b)** active minutes, and **(c)** sedentary hours plotted against gestational age for all 49 participants. Each dot represents the mean steps, active minutes, or sedentary hours for any participant who had ATD data during the corresponding gestational week. The blue line, or LOESS curve, is a nonparametric method for fitting a curve through points on a scatter plot, revealing trends in the data.

**FIG. 4.**
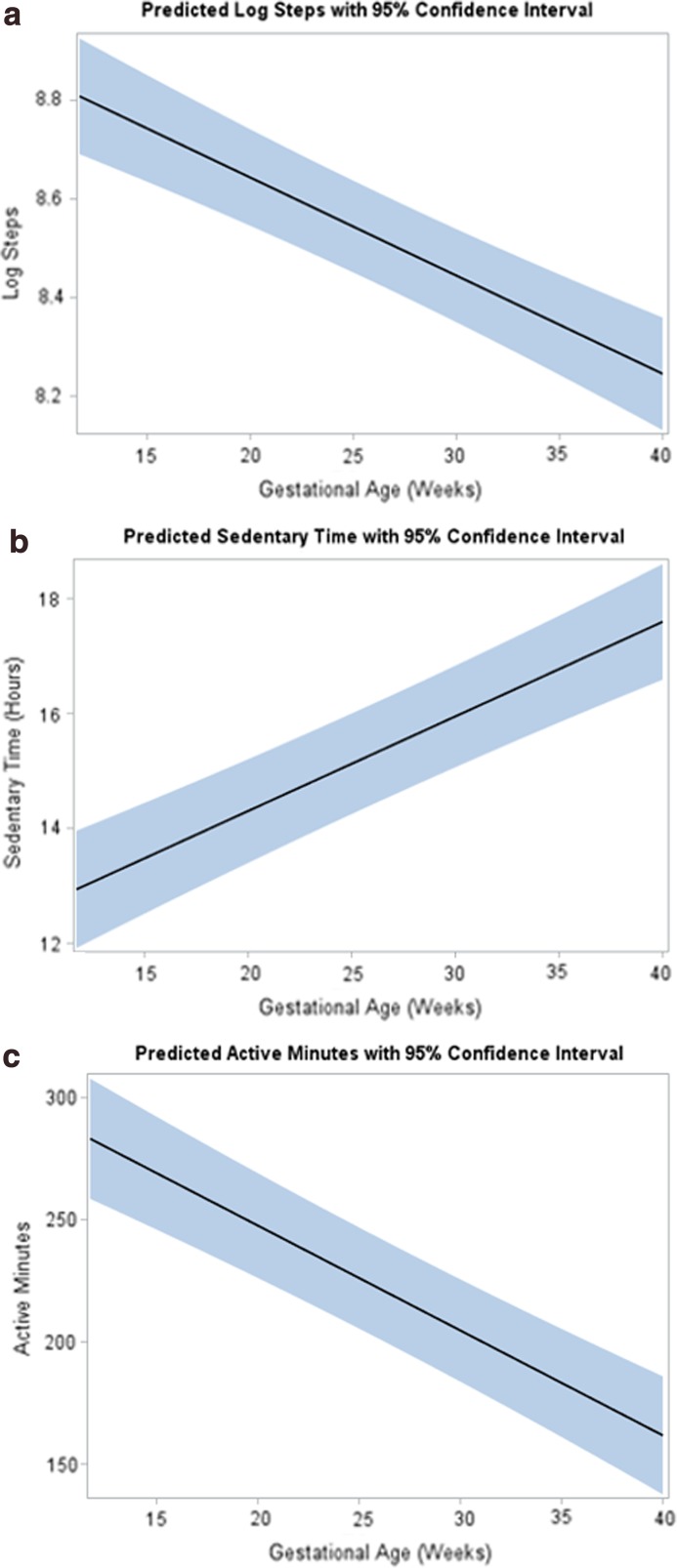
Longitudinal modeling for ATD data with predicted **(a)** logarithmic steps, **(b)** sedentary hours, and **(c)** active minutes as denoted by black lines with 95% CI (shaded area) plotted against gestational age with *p* < 0.001 for change over time for all comparisons.

Figure 3 presents the mean steps, active minutes, and sedentary hours per day by gestational age for all 49 participants. Longitudinal models indicated that as gestational age increased, mean log steps (β = −0.02, *p*-value <0.001, Fig. 4a) decreased, mean active minutes decreased (β = −4.37, *p*-value <0.001, Fig. 4b), and mean sedentary hours increased (β = 0.17, *p*-value <0.001, Fig. 4c). These differences persisted after adjusting for age, ethnicity, prepregnancy BMI category, and education. There were no significant differences in mean energy expenditure (MET-h/week) recorded by PPAQ or ATD data at 28 weeks [231 (62–927 range) vs. 238 (212–290 range), *p*-value = 0.74] and at 36 weeks [145 (35–581 range) vs. 222 (196–272 range), *p* = 0.27], but there were differences in the variances of these measures at both 28 and 36 weeks (*p* < 0.001).

## Discussion

In this feasibility study of ATD use in group prenatal care, most participants reported satisfaction with respect to wearing the ATD and the information it provided and would recommend the ATD to others. However, adherence to the study protocol was low, with less than half of participants who completed the final study survey self-reporting daily ATD wear by the end of pregnancy. Furthermore, according to the ATD data, participants wore the ATD only 43.7% of possible days.

These findings deviated from our original hypothesis of participants wearing the ATD for more than 80% of the time from enrollment until delivery and less than 10% of participants reporting major issues or technical difficulties with the ATD. Some of the reported adherence issues were related to lost or broken ATD or charger, syncing issues, and loss of the participants' own cell phone or wireless network, affecting more than 25% of the initial 49 participants.

Our findings have similarities and differences compared with other reports of ATD use in the general population. Macridis et al. used a telephone interview to examine the prevalence and use of ATD in 1215 adults in Alberta, Canada. In their sample, which comprised 50% women and 60% overweight or obese participants, 35.5% owned an ATD and only 19.6% currently were using their ATD. ATD users wore it for an average of 23 days in the month preceding the survey.^29^ The participants who owned an ATD, but who no longer used it, reported they wore the ATD for ∼8 months before they stopped wearing it altogether. Another report from a national cross-sectional survey found that 37% used an ATD for 1 month, 35% for 1–6 months, and 27% for more than 1 month.^30^ Pregnancy status was not mentioned in either of these two studies.

Few studies have reported on ATD use in pregnancy. Huberty et al. gave 80 inactive pregnant women (defined as reporting less than 30 min of moderate physical activity on at least 4 days/week) an ATD at 8–16 weeks of gestation and found similar trajectory patterns in activity with an overall decline in activity and increase in sedentary time as gestational age increased.^28^ Women wore the ATD for an average of more than 100 days throughout their pregnancy and took more than 4000 steps/day. Based on adjusted models, women spent ∼16 h being sedentary in the midtrimester regardless of their BMI group, which is higher than our values (mean 12 h; range 7.8–16.2 for the first week of use).^28^

It was interesting to find that self-reported activity via the PPAQ did not decline as gestational age increased, as it did for the ATD step counts, especially since 70% of women reported that they were exercising less-often at the final survey compared with before pregnancy. Prepregnancy exercise levels were considered in adjusted longitudinal models for log total steps, sedentary time, and active minutes. In all models, prepregnancy exercise was not significantly associated with the outcome, and had very little effect on the size or significance of the gestational age estimates.

Furthermore, the comparison of mean MET-h/week by reporting tool (self-report via PPAQ vs. ATD data) did not indicate a statistically significant difference at any time point; however, we did observe a much greater variation in PPAQ-reported METs compared with ATD-recorded METs across participants. Both positive and negative differences between PPAQ and ATD METs could indicate both underestimation and overestimation from self-reported PPAQ measures. The average absolute difference between a reported PPAQ and ATD-recorded MET-h/week over the second and third trimesters was 125.3 MET-h/week, with absolute differences as large as 705 MET-h/week, suggesting potential inaccuracy of the PPAQ measure.

We are not aware of other studies that compared self-reported vs. ATD data energy expenditure. Due to the lack of recall bias with ATD, we suspect that the ATD has the potential to have greater accuracy, but further studies are indicated to evaluate the differences between self-reported and ATD-calculated energy expenditure.

Our ATD data confirm what we know from prior studies of population-level physical activity in pregnancy, including a decrease in activity and increase in sedentary time as gestational age increases, yet the ATD now allows us to monitor activity in a more contemporary manner. We opted to study ATD use among participants in group prenatal care, where social support might also motivate women to reach pregnancy goals, including physical activity. However, the findings that participants did not engage in the community forums are similar to those of Macridis et al. who reported that 51% of their participants did not find connecting with friends/family for step challenges useful.^29^ To protect participant confidentiality, no identifying information was posted to the community forums. Lack of identity may have diminished the attractiveness of connecting with other pregnant women in this manner.

A notable proportion of women who consented to the study did not ultimately use the ATD according to the intended protocol. Future trials could consider the option of intermittent use at selected intervals to improve compliance. Further study is also needed to evaluate how physical activity changes after delivery, given that many women weigh more than their prepregnancy weight 1 year after delivery and encounter additional barriers to health behavior changes.^31^ Although it was required for all women to have their own cell phone for participation, individual cell phone plans and their payments were not offered in this study. This was an issue for at least three participants who reported losing their cell phone and/or cell phone account.

The wrist location for the ATD was specifically chosen due to the physiological changes that were predicted to create difficulty with a waist ATD in pregnancy. Still, participants reported not wearing the wrist ATD due to edema, even though participants had the option of wearing two different band sizes. Furthermore, the wrist location may have overestimated activity that involved only upper and not lower extremity motions,^32^ although another study found moderate to high correlations with hip and wrist-worn accelerometers in 100 pregnant and postpartum women.^33^ Finally, we used the manufacturer-provided information regarding activity levels and METs (“very active minutes,” “fairly active minutes,” and “lightly active minutes”), yet we realize specific cut points for these levels have not been determined in pregnant women.

Participants who returned the ATD after enrollment reported that it “did not fit (their) lifestyle” or that they had too many other events going on in their life to focus on physical activity. These reports underscore the challenges women face when attempting to make lifestyle changes. Furthermore, participants may have preconceived notions about potential risks of exercise in pregnancy and/or receive advice from other support systems, given that 50% of participants disagreed with the statement, “Physical activity that makes me breathe harder is ok at any time during pregnancy.”

We acknowledge limitations to this study, including the small sample size and inability to critically evaluate several important pregnancy outcomes, such as GWG and birthweight, as a function of ATD data. We also acknowledge that no corrections were made for multiple hypothesis tests, and thus, significant results should be interpreted with caution. We opted to use the Fitbit model for our ATD because it had the highest rating for validity in other studies, but to our knowledge, there has been no formal validity testing of Fitbits in pregnant women.^34^

## Conclusion

Self-monitoring of physical activity may increase awareness of one's activity level and consequently lead to behavioral change necessary to meet physical activity goals during pregnancy. There is still a need for future research to investigate barriers and facilitators to ATD use in pregnancy, especially as the technology for ATD and smartphone apps rapidly changes. Given that pregnancy is viewed as a window of opportunity to influence current and future health behaviors, finding newer ways to engage women in physical activity and reduce sedentary behaviors in pregnancy is a research priority.

## Abbreviations Used

ATD: activity tracking device
BMI: body mass index
GWG: gestational weight gain
IQR: interquartile range
METs: metabolic equivalents
PPAQ: Pregnancy Physical Activity Questionnaire
